# Inhibition of *Pseudomonas aeruginosa* biofilm formation on copper-based thin foils

**DOI:** 10.1371/journal.pone.0314684

**Published:** 2024-12-05

**Authors:** Andrea Timoncini, Luca Lorenzetti, Raymond J. Turner, Ashley McGibbon, Carla Martini, Elena Cofini, Elena Bernardi, Cristina Chiavari

**Affiliations:** 1 Department of Pharmacy and Biotechnology, University of Bologna, Bologna, Italy; 2 Department of Industrial Engineering, University of Bologna, Bologna, Italy; 3 Department of Biological Sciences, University of Calgary, Calgary, AB, Canada; 4 Department of Biomedical Sciences, McGill University, Montreal, QC, Canada; 5 Department of Cultural Heritage, University of Bologna, Ravenna, Italy; 6 Department of Industrial Chemistry “Toso Montanari”, University of Bologna, Bologna, Italy; Laurentian University, CANADA

## Abstract

The development of Healthcare-Associated Infections (HAIs) represents an increasing threat to patient health. In this context, *Pseudomonas aeruginosa* is responsible for various HAIs, determining about 20% of the infections in hospitalized patients, which makes it one of the most effective pathogens due to its strong ability to form biofilms. Using Cu-based materials as foils on high-touch surfaces can help to prevent and mitigate *P*. *aeruginosa* contamination in biohazardous settings. However, the antibiofilm properties of Cu-based surfaces against *P*. *aeruginosa* may vary due to frequent touches combined with indoor environmental exposure. The main aim of this study is to investigate the impact of accelerated ageing, mimicking a high-touch frequency by cyclic exposure to artificial sweat solution as well as to temperature and relative humidity variations, on the efficacy of Cu-based thin foils against *P*. *aeruginosa* biofilms. Three Cu-based materials (rolled and annealed Phosphorous High-Conductivity (PHC) Cu, Cu15Zn brass, and Cu18Ni20Zn nickel silver) were evaluated. The ageing process enhanced the antibiofilm properties, due to an increment in Cu ion release: aged PHC Cu and Cu15Zn exhibited the highest Cu ion release and hence the highest biofilm inhibition (decrease in colony forming unit (CFU)) in comparison to their pristine counterpart, while aged Cu18Ni20Zn displayed the lowest biofilm formation reduction, despite showing the highest aesthetic and morphological stability. The Cu-based surface, which highlited the highest biofilm formation inhibition due to accelerated ageing, was Cu15Zn.

## 1 Introduction

The presence of microbial cells on surfaces in healthcare facilities poses a significant threat to patient health and imposes substantial economic burdens due to the development of Healthcare-Associated Infections (HAIs). It is estimated that at least 5 million HAIs annually occur in acute care hospitals across Europe, resulting in approximately 135000 deaths per year. These infections account for around 25 million additional hospital days, with an associated financial burden ranging from €13 billion to €24 billion [1].

*Pseudomonas aeruginosa* is responsible for various HAIs [2]. It is classified as a critical priority pathogen of serious concern due to its high pathogenicity level, which is due to multidrug resistance (MDR), adaptability (i.e., quorum sensing (QS)) and virulence factors (i.e., biofilm formation) [3–5]. *Pseudomonas aeruginosa* PAO1 is an opportunistic microorganism involved in shaping biofilms associated with human and veterinary cutaneous wound and lung infections of patients with cystic fibrosis, otitis and bacterial endocarditis [6, 7]. Among individuals who are critically ill or immunocompromised, it is the primary cause of bacteremia and pneumonia, as well as urinary tract, catheter-associated and skin/soft tissue infections [8, 9]. This species shows increasing frequency in MDR, which poses significant threats in nosocomial settings due to its ability to cause highly severe infections [2] and since new antibiotic classes could be ineffective against it [10]. This pathogen can be widespread in hospitals and nursing homes via attachment, establishing colonies and thus developing biofilm on surfaces. A biofilm has been defined as a complex microbial consortium wherein cells are bound together through an extracellular matrix (ECM) generated by the cells [7]. ECM and cells are crucial for adhering to the substratum [11]. *P*. *aeruginosa* exhibits a high capacity for biofilm development, as it contains eight penicillin-binding proteins which play a crucial role in biofilm formation and maturation [7], in addition to (QS) mechanisms that regulate biofilm structure [12, 13]. The presence of biofilms accounts for up to 65% of all (HAIs), with *P*. *aeruginosa* responsible for approximately 20% of infections in hospitalised patients, particularly affecting those who are immunosuppressed [7]. The critical surfaces (fomites) encompass various items such as bed rails, health instruments, furniture, and medical indwelling devices in both hospital rooms and Intensive Care Units (ICUs) [7]. Bacteria generally prefer wet surfaces, where their survival may be higher than on dry ones [14]. However, they can endure extended periods on desiccated surfaces. Biofilm development is also fundamental for reducing sensitivity to antimicrobial drugs [9], biocides, antibiotics, physical stressors and harsh environmental conditions [15]. Therefore, prioritising infection control and disease prevention measures is crucial to mitigate the dissemination of MDR pathogens [7, 10]. Transition metals such as copper (Cu) exhibit efficacy against microbes which grow within a biofilm structure [16, 17].

Cu exerts antimicrobial action in both wet and dry conditions. In the former (i.e., in suspension or culture), free Cu ions are responsible for bacterial growth arrest or killing primarily via iron replacement in iron-sulfur cluster proteins, followed by Reactive Oxygen Species (ROS) production [18]. On dry surfaces, the process is known as "contact-killing”, which occurs within minutes to hours [19, 20]. The exact mechanisms underlying "contact-killing" and microbial interaction with released ions remain only partially understood. However, it is accepted that Cu release from surfaces results in the accumulation of millimolar (mM) concentrations within the thin aqueous film typically present even on apparently “dry” metallic surfaces. Subsequently, cell interaction with the surface leads to an influx of Cu ions. This overwhelms the cells’ Cu buffering systems, leading to Cu replacing other metal ions in enzymes with the most metabolic activities inhibited. Genomic and plasmid DNA degradation occurs through unknown mechanisms, perhaps through ROS, as Cu can also catalyse Fenton reactions. Eventually, exposure to metallic Cu typically results in the inability to detect structurally intact bacteria [18]. For this reason, implementing Cu on fomites in hospital equipment, as surface or coating, decreases microbial burden, mitigates the risk of HAIs [21–25] and decreases treatment expenses [26]. The use of Cu alloy surfaces has been demonstrated to reduce ICUs bacterial amount by 83% after 23 months of exposure and to reduce the frequency of HAIs by 56% [21, 22].

Utilizing thin foils based on Cu alloys applied onto existing surfaces represents a rapid, straightforward, and economically viable method for contamination prevention. This approach circumvents challenges associated with the substantial expenses of bulk metal supply, reducing the necessity for newly manufactured components. However, the alloy composition and exposure to indoor environments can alter a Cu-based surface’s antimicrobial efficiency and in-service durability [27]. Also, surface roughness, a key factor influencing both wettability and microbial persistence [28], can exhibit variation under repeated indoor contact, alongside colour and aesthetic properties.

Previous studies investigated the antimicrobial properties of Cu-based surfaces exposed to artificial sweat under general indoor atmospheric conditions [19, 29], but the influence of conditions specific to healthcare settings on the ageing of Cu-based freestanding foils has not been fully investigated yet. Therefore, this study investigated the effect of accelerated ageing, which simulates humidity and temperature conditions in healthcare environments, on the morphological, aesthetic and antibiofilm properties of Cu-based thin foils produced via industrial manufacturing processes. The influence of alloy composition and microstructure of Cu-based surfaces on *P*. *aeruginosa* biofilm inhibition was evaluated over the ageing process. Cu release in Luria-Bertani (LB) solution was used to investigate the antibiofilm properties of the Cu-based foils (15–25 μm thick), which consisted of Phosphorus High-Conductivity (PHC) Cu (99.95% wt—benchmark), Cu15Zn, and Cu18Ni20Zn. Moreover, for PHC Cu foils, the effect of a variation in the manufacturing cycle (with or without final annealing, which induces recrystallisation and relieves strain hardening generated by cold rolling) was investigated, because such a variation affects the microstructure and hence Cu release.

## 2 Materials & methods

### 2.1 Materials

The following alloy compositions characterised the investigated Cu-based freestanding foils:

1. Phosphorous High-Conductivity PHC Cu (Cu: 99.95 wt.%);
2. Cu15Zn (brass 85/15);
3. Cu18Ni20Zn (nickel silver 62/18).

The chemical composition of the thin rolled foils was checked before accelerated ageing by Glow Discharge Optical Emission Spectroscopy (GD-OES) with a Grimm-style glow discharge lamp operating in DC mode (Spectruma Analitik GDA 650).

Pure Cu, Cu15Zn and Cu18Ni20Zn have already been listed as public health anticontamination products by EPA [30]. Cu15Zn and Cu18Ni20Zn exhibit enhanced mechanical properties [31] and corrosion resistance compared to pure Cu [32].

Cu-based thin foils were produced in an industrial environment (Pietro Galliani SpA, Italy) by a comparable manufacturing cycle (Table 1), and processing conditions were customised for each alloy to achieve a thickness in the range of 15–25 μm, making the Cu-based foils ready for applications on high-touch surfaces.

**Table 1:** Cu-based foils: CID (UNS), manufacturing cycle and foil thickness.

CID (UNS)	Label	Thermo-mechanical treatments	Foil thickness (μm)
C10300	Cu R	Cold rolling	24.5 ± 0.6
	Cu R+A	Cold rolling + final annealing (670°C)	24.1 ± 0.6
C23000	Cu15Zn		27.0 ± 0.3
C75200	Cu18Ni20Zn	Cold rolling (with intermediate annealing (720°C)) + final annealing (720°C)	12.6 ± 0.2

The manufacturing cycle involved rolling with a subsequent final annealing step. Pure Cu was provided after being subjected to both rolling (henceforward Cu R) and rolling followed by annealing (Cu R+A) to assess the influence of annealing on microstructure and biofilm formation.

**Fig 1:**
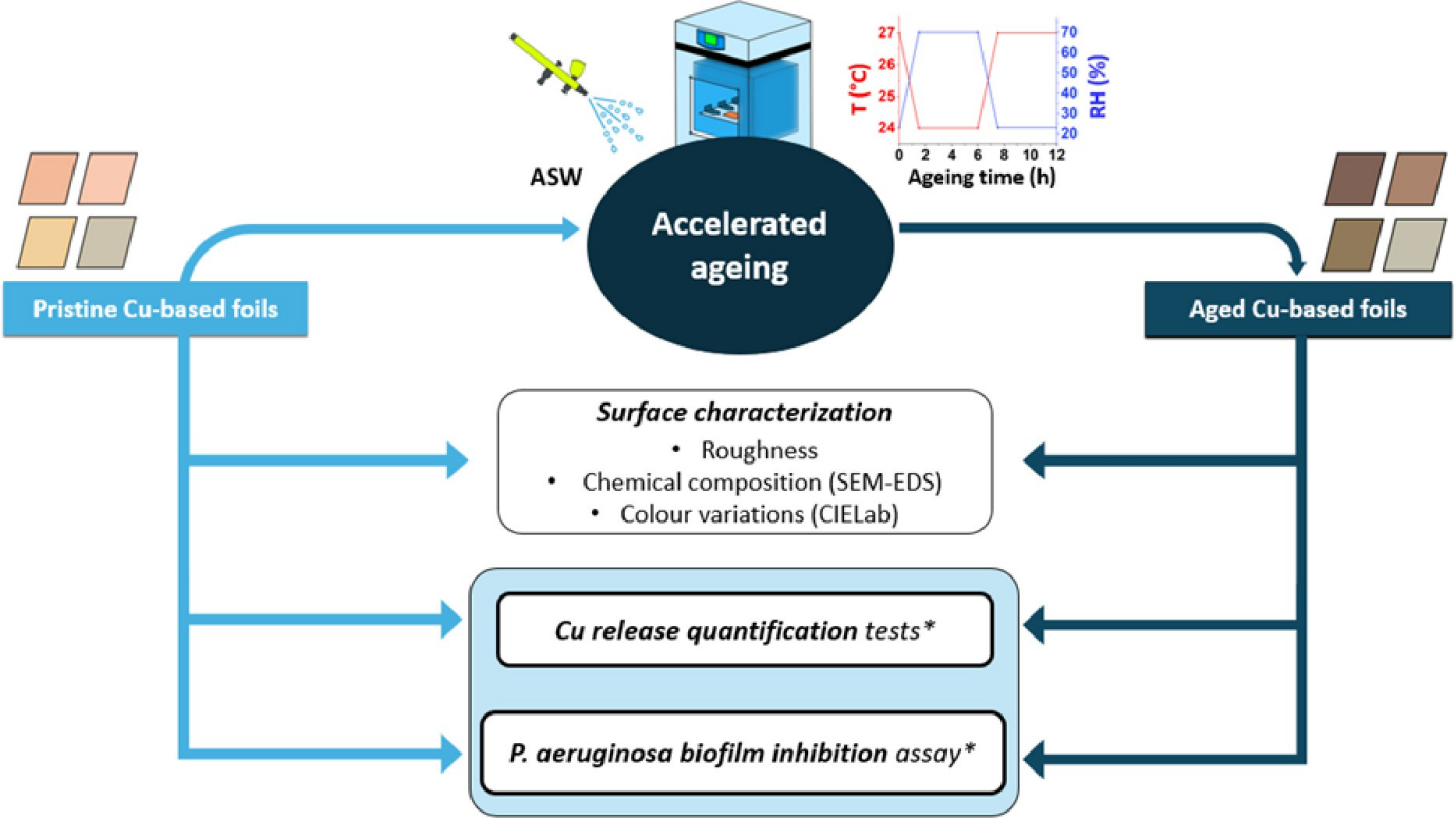
Flow chart of the methodology utilized in this study. * stands for Luria Bertani (LB) broth.

The workflow of this study is presented in Fig 1. It involves applying the same methodological procedure to both pristine and aged surfaces. The ageing process, necessary to get the aged Cu-based foils, is first explained. It follows the surface characterisation, including roughness, chemical composition and colour variations. The core of this research is the Cu release and *P*. *aeruginosa* biofilm inhibition assay.

### 2.2 Accelerated ageing of thin Cu-based foils simulating human touch conditions in hospital settings

An artificial human perspiration solution was utilized to mimic the tarnishing induced by repeated hand touch. Fingerprint residue was simulated by nebulizing droplets of artificial sweat solution (ASW, EN1811 [33]) onto each Cu-based surface. ASW was prepared using 5.0 g/L NaCl, 1.0 g/L urea, and 1.0 g/L lactic acid dissolved in ultrapure water (Milli-Q®, ρ < 18 MΩ). The pH of the solution was adjusted to 6.5 ± 0.05 with NaOH. Freshly prepared ASW was used for all experiments.

The spraying method yielded a reproducible distribution of small droplets across the surface, with a deposited mass of 2.59 ± 0.06 mg_ASW_/cm² (averaged over 5 repetitions). Previous studies have reported an average palmar sweat rate in humans of approximately 0.1 mg/cm² [34]. Therefore, the deposited mass per cycle in this study corresponds to approximately 26 human touches per cycle. In hospital settings, bedside tables represent surfaces with the highest contact frequency, experiencing an average contact rate of approximately 14 contacts per hour [35]. Hence, in this study, each spray of ASW simulates contact exposure of approximately 2 hours in a real-world context.

**Fig 2:**
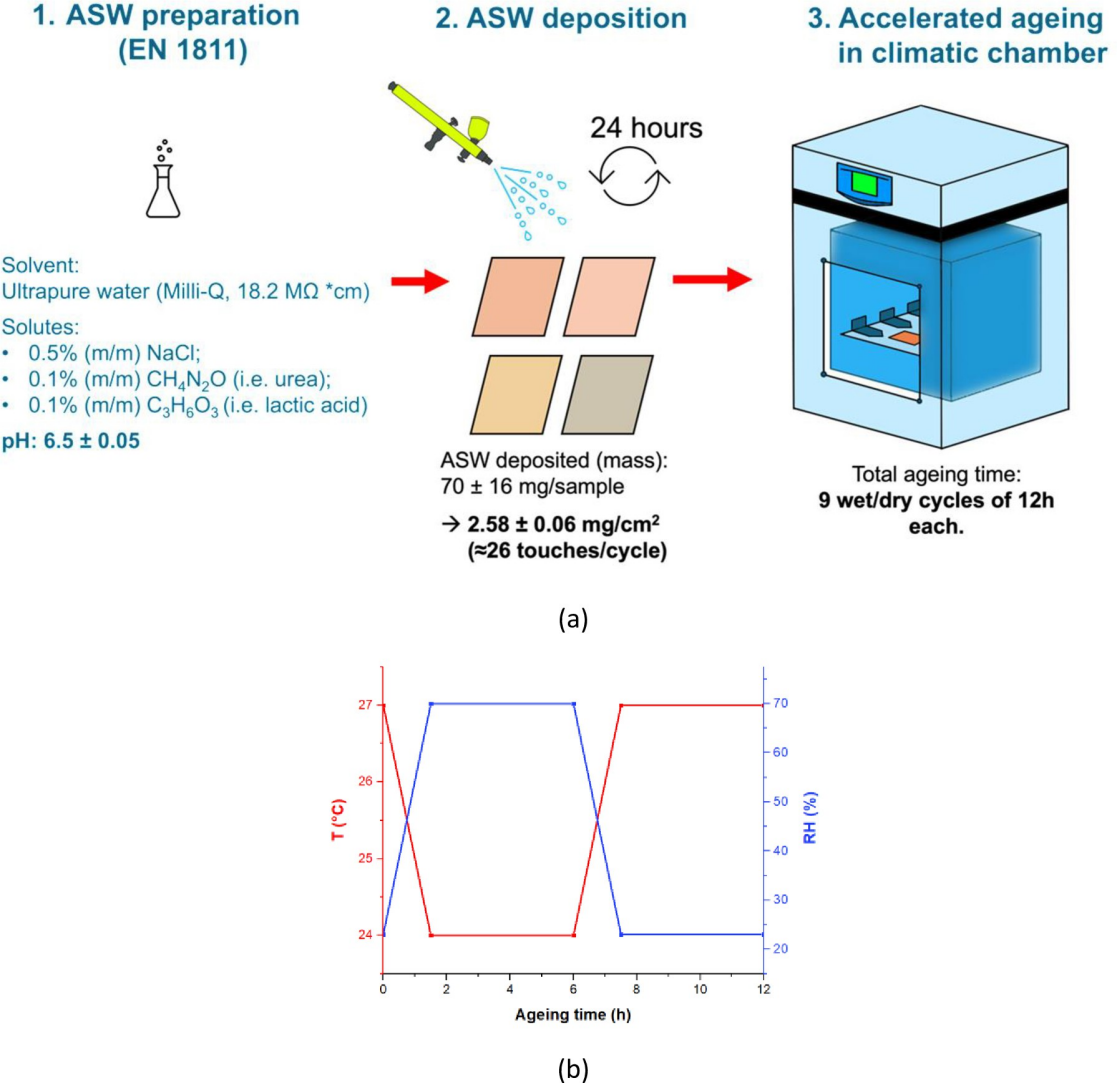
(a) Schematic of sample preparation and (b) Temperature (°C) and relative humidity (%) during a unit cycle (12 hours) of wet-dry laboratory exposure within a climate chamber.

Accelerated ageing was performed using a modified version of the protocol used by Chang *et al*. [19]. Fig 2 shows a schematic of the ageing methodology.

Cu-based surfaces, pre-sprayed and daily-sprayed with ASW were placed into a climatic chamber (Fig 2A) and subjected to a cyclic wet/dry atmosphere (Fig 2B), replicating indoor environment conditions. Each cycle comprised 12 hrs: two wet/dry phases separated by intermediate transition stages (i.e., 1.5 hrs). The wet/dry phases were: (i) 4.5 hrs at RH 70% and 24°C, followed by (ii) 4.5 hrs at RH 23% and 27°C. Ageing lasted for 108 hrs in total. The indoor environmental parameters for each cycle were chosen to mimic variations in temperature and relative humidity typically recorded during a year in hospital environments [36–39].

Surfaces that were not subjected to artificial ageing will be designated as "pristine", whilst surfaces that underwent artificial ageing will be referred to as "aged".

Each sample was weighed using an analytical balance (with an accuracy of 0.0001 g) before and after accelerating ageing to evaluate mass variations due to exposure.

### 2.3 Surface characterization before and after accelerated ageing

Surface morphology was observed before and after ageing by optical (Hirox KH 7700) and scanning electron microscopy (Zeiss EVO 50) at an accelerating voltage of 10 kV. Variations in chemical composition due to ageing were evaluated by Energy-Dispersive Spectroscopy (EDS, Brucker Quantax 200/30 mm^2^) by averaging values from 3 macro-areas of 1.1. mm^2^ for each sample.

Colour variations after ageing were quantitatively assessed by reflectance spectrophotometry (3NH-YS3060, with d/8 geometry, D65 illuminant, 10° observation angle, 8 mm measurement area and specularity included). The measurements were carried out in the CIELab color space [40], where the main coordinates are a* (red/magenta-green), b* (yellow-blue) and L* (lightness–black to white), while the total color variation is given by:

$$\Delta \text{E}=\sqrt{{\left(\Delta L^{*}\right)}^{2}+{\left(\Delta a^{*}\right)}^{2}+{\left(\Delta b^{*}\right)}^{2}}$$

The roughness of free surfaces was measured by non-contact 3D profilometry (Nanovea JR25) using Lt = 4 mm, Lc = 0.8 mm according to ISO 4288–1998, with scanning step: 2 μm, acquisition rate: 400 Hz. The surface topography was characterized by mapping a 2x2 mm^2^ area. For these measurements, scanning steps of 2 μm, averaging mode of 5 and dual frequency were used (i.e., 400 and 1850 Hz).

### 2.4 Evaluation of *Pseudomonas aeruginosa* attachment on different Cu alloy surfaces

The strain of *P*. *aeruginosa* used was the American Type culture collection strain named *Pseudomonas aeruginosa ATCC2785*, stored at −70°C at the University of Calgary, Canada. The evaluation of *P*. *aeruginosa* biofilm inhibition on Cu-based surfaces was carried out by diluting an overnight (O/N) culture of *P*. *aeruginosa*, grown in Luria-Bertani (LB) media to get an Optical Density reading of 0.8 at OD_600_, which corresponds to c.a. 10^8^ bacterial cells/mL. The O/N culture inoculum came from the stock transferred on LB agar plates to grow O/N at 37°C, and then moved in a test tube with 2 mL of LB media and grown O/N at 37°C.

The cell density was determined from the absorbance/light scattering at 600 nm using a UV-visible spectroscopy (Thermo Scientific GENESYS™ 30 Visible Light Spectrophotometer) as a proxy for cell numbers as in regular culturing practice [41].

Subsequently, 10 μL of culture solution were deposited onto each metal coupon. Before being exposed to bacteria culture, each coupon was cut to obtain a circular area of 1 cm^2^ and sterilized in ethanol 96% and air dried. After 30 min of bacterial exposure each sample was washed twice in PBS solution in two consecutive trays, then each sample was placed into 1 mL of LB solution and sonicated to detach the adherent cells. The released bacterial cells were counted by taking 10 mL of surrounding media at the end of the experiment, serially diluted 10-fold to reach the 10^−8^ dilution, followed by spot plating on the LB agar plate from bottom to top and incubated for 26 hrs where the cell number was counted. The CFU/mL value was obtained by the eqn. proposed by Hazan *et al*. [42]:

(CFUmL)=numberofcolonies*100*10-dilution

**Fig 3:**
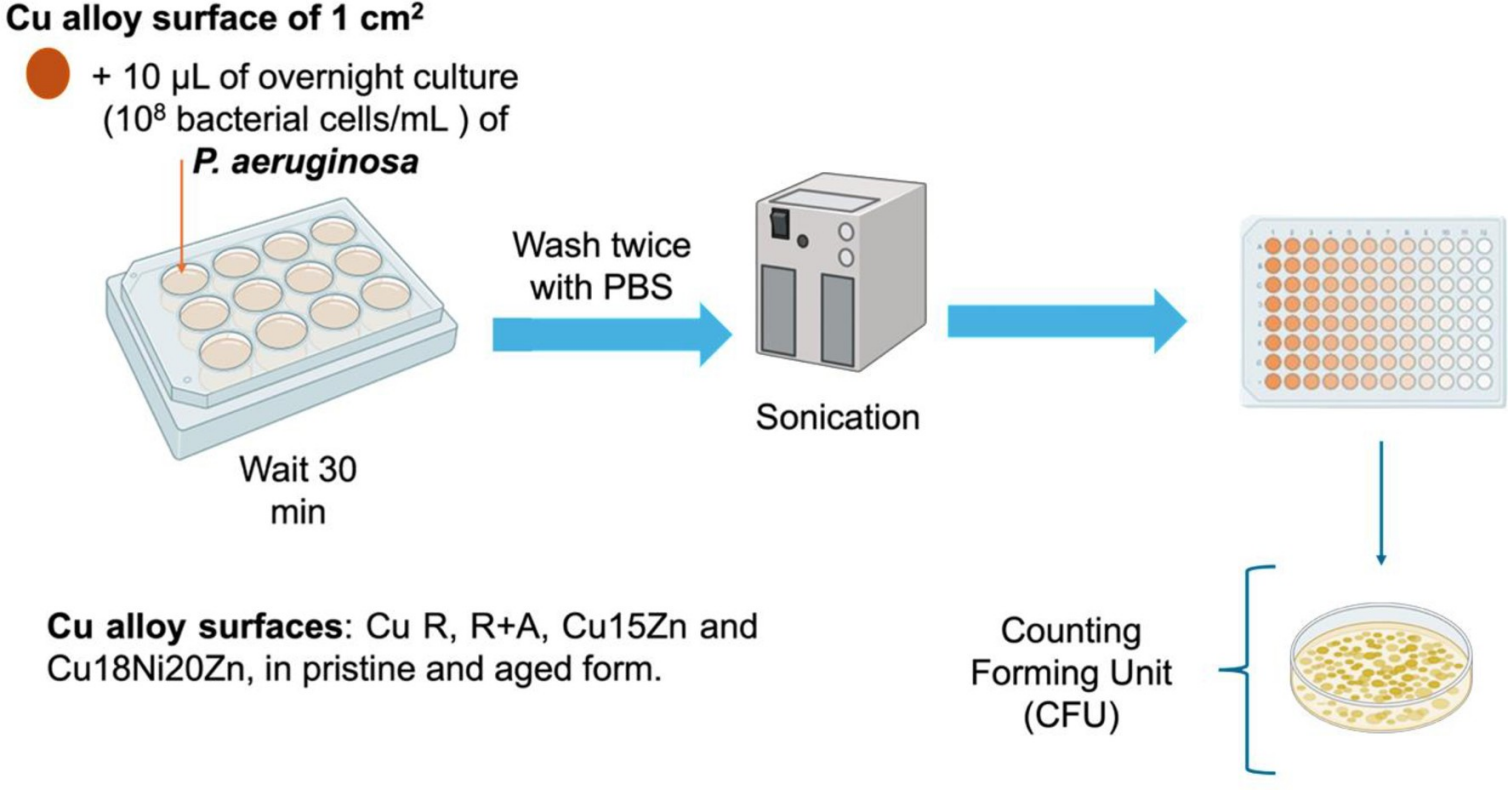
Workflow for the evaluation of inhibition of biofilm formation by *P*. *aeruginosa* on Cu alloy surfaces.

CFU/mL values were normalized per drop area (cm^2^) and were reported in Log_10_ scale. For each pristine substrate the area was 0.0830 cm^2^, while for the aged substrates, it was 0.1963 cm^2^ except for aged Cu18Ni20Zn (0.2827 cm^2^). The experiment was carried out with two technical replicates within two biological replicates (workflow in Fig 3).

### 2.5 Evaluation of Cu release in LB

**Fig 4:**
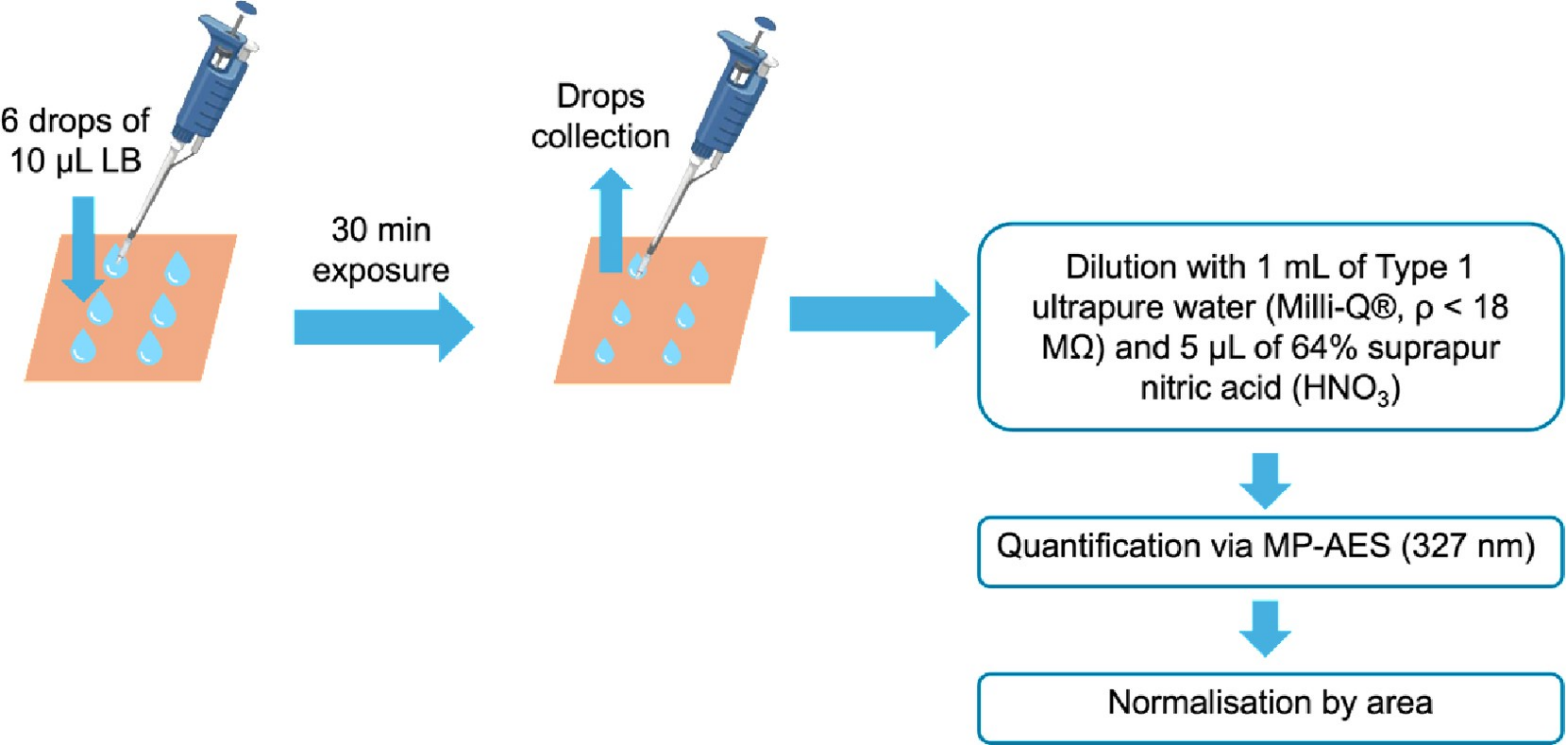
Process of Cu release quantification.

Cu release was quantified by adapting the *quasi-dry* protocol developed by Chang *et al*. [19], using LB without dispersed pathogens as the testing solution: 6 droplets (10 μL each) were deposited onto each Cu-based foil, as shown in Fig 4.

All the 6 LB droplets were in contact with the surface foils for 30 minutes before being collected and stored in a 4.5 mL polystyrene tube. 3 replicas were carried out for each substrate and condition (i.e. pristine and aged). After collection, the volume of LB droplets was checked to account for the effect of evaporation. The samples were then diluted with 1 mL of Type 1 ultrapure water (Milli-Q®, ρ < 18 MΩ), and 5 μL of 64% suprapur nitric acid (HNO_3_) was added to prevent redeposition of dissolved Cu ions. The samples were maintained at 2–4°C before quantification analysis. 30 minutes was selected as the exposure time in relation to the antimicrobial testing conditions.

Total dissolved Cu was quantified by Agilent Technologies 421 Microwave Plasma Atomic Emission Spectroscopy (MP-AES), encompassing both Cu(II) and Cu(I) cations as well as any dissolved Cu complexes. Calibration standards of Cu were prepared using concentrations of 0.5; 1; 2.5; 5; 20; and 30 ppm. Cu release was quantified using the peak intensity at the wavelength of 327.39 nm. At the same time, blank reference solutions (LB only) were examined and used to correct Cu background concentrations for each condition.

Area measurements were carried out to normalise Cu release by depositing one droplet of 10 μL on each surface and estimating the contact area based on optical microscopy images processed by ImageJ. The measures were performed in duplicate.

For descriptive statistics of normally distributed data sets, the mean ± standard error was utilised to summarize measurements of Cu release (μg/cm^2^). For intra- and inter-group comparisons of Cu release, analysis of variance (ANOVA) was employed, and, where a statistically significant difference was found, all pairwise multiple comparisons were conducted using the Holm-Sidak test, provided that all data sets were normally distributed. The t-test was applied to investigate statistical differences between pristine and aged for the same substrate type. Each statistical test has been performed on 3 independent replicates, with alpha = 0.05. All statistical analyses were conducted in the SigmaPlot 11.0 environment.

## 3 Results & discussion

### 3.1 Surface characterization before and after accelerated ageing

The chemical composition of each Cu-based material is detailed in Table 2, alongside nominal data for the alloy types with the closest composition.

**Table 2:** Chemical composition of Cu-based foils (wt.%): Experimental results obtained via GD-OES compared to nominal compositions [43].

	Elements (wt %)				
	Cu	Zn	Ni	Fe	Others
Cu PHC	99.74 ± 0.02	0.050 ± 0.002	-	0.21 ± 0.02	-
*Nominal*	*99*.*95*	*-*	*-*	*-*	*0*.*001–0*.*005*
Cu15Zn	85.33 ± 0.80	14.42 ± 0.67	-	-	0.25
*Nominal*	*84–86*	*14–16*	*-*	*0*.*05*	*0*.*05*
Cu18Ni20Zn	60.23 ± 0.20	20.74 ± 0.13	18.15 ± 0.13	0.023 ± 0.001	0.86
*Nominal*	*63*.*0–66*.*5*	*16*.*5–19*.*5*	*16*.*5–19*.*5*	*0*.*25*	*-*

**Fig 5:**
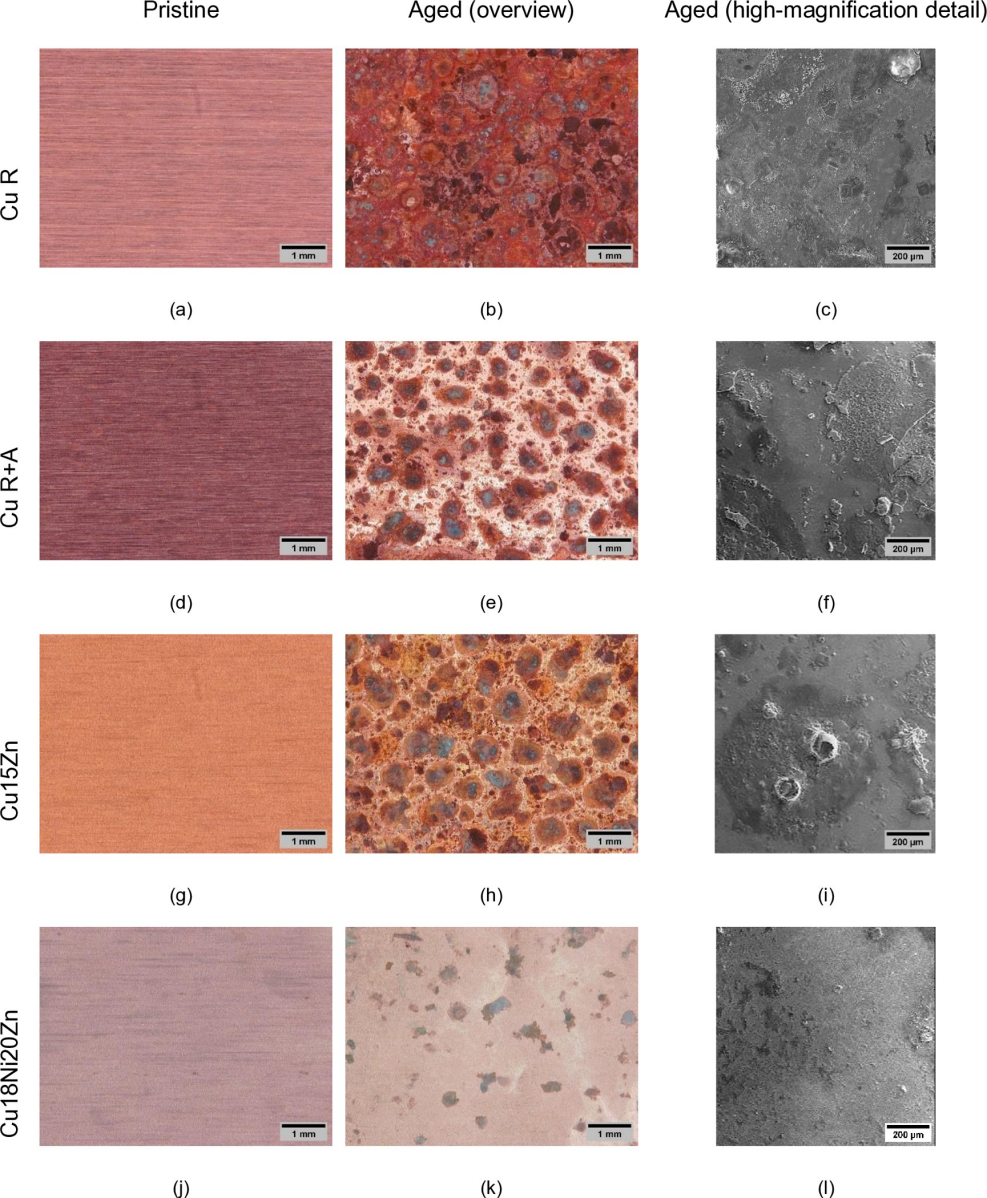
Optical images before (a, d, g, j) and after ageing (b, e, h, k), with high magnification details of aged surfaces (secondary electron images: c, f, i, l).

Surface morphology (Fig 5) and chemical composition (Table 3) reflect variations before and after accelerated ageing.

**Table 3:** SEM-EDS analysis of the Cu-based foils before and after accelerated ageing (1.1 mm^2^ areas) and mass variation (Δm%) due to ageing.

		Elements (wt %)								Δm %
Surface	Type	Cu	Zn	Ni	O	N	Na	Cl	C	
**Cu R**	**Pristine**	95.8 ± 0.4	n.d.	n.d.	0.7 ± 0.1	0.2 ± 0.2	n.d.	0.03 ± 0.02	3.3 ± 0.4	3.4
	**Aged**	66.1 ± 5.9	n.d.	n.d.	17.1 ± 2.3	0.8 ± 0.3	2.5 ± 2.8	5.1 ± 2.6	8.5 ± 1.1	
**Cu R+A**	**Pristine**	95.2 ± 0.2	n.d.	n.d.	0.6 ± 0.1	0.3 ± 0.1	n.d.	0.03 ± 0.03	3.7 ± 0.3	3.2
	**Aged**	60.5 ± 16.7	n.d.	n.d.	15.1 ± 5.9	1.0 ± 0.2	10.9 ± 10.8	4.0 ± 1.7	8.2 ± 1.1	
**Cu15Zn**	**Pristine**	76.7 ± 0.3	18.2 ± 0.3	n.d.	2.3 ± 0.1	0.01 ± 0.01	n.d.	0.01 ± 0.01	2.7 ± 0.3	1.3
	**Aged**	52.1 ± 4.5	7.3 ± 0.8	n.d.	16.6 ± 4.7	0.6 ± 0.2	10.3 ± 1.1	4.9 ± 0.5	8.3 ± 0.6	
**Cu18Ni20Zn**	**Pristine**	63.4 ± 0.4	18.1 ± 0.3	12.9 ± 0.2	1.10 ± 0.03	0.44 ± 0.03	n.d.	n.d.	4.0 ± 0.6	+4.1
	**Aged**	38.4 ± 7.4	12.5 ± 3.0	10.9 ± 2.2	8.6 ± 3.7	0.6 ± 0.5	13.4 ± 6.3	7.1 ± 5.3	8.5 ± 1.0	

n.d.: not detectable

Localized corrosion occurred on all the surfaces, due to the ageing sequence, involving cyclic changes in humidity and temperature, combined with daily spraying of ASW. Surface morphology (Fig 5) and elemental composition (Table 3) variations evidenced the formation of corrosion products. Table 3 shows a notable increase in O %, Na % and Cl % wt. demonstrating the formation of surface oxides and chlorides and/or more complex corrosion products, which contributed to the mass increase after ageing (Table 3). The concentration of C, N, Na and Cl, adventitious (in the case of pristine samples) and/or deriving from residual ASW solution components (for aged samples) always increased during ageing. These elements, together with oxygen, can become part of the corrosion products or be present on the surface as an unreacted deposit of ASW after drying.

**Fig 6:**
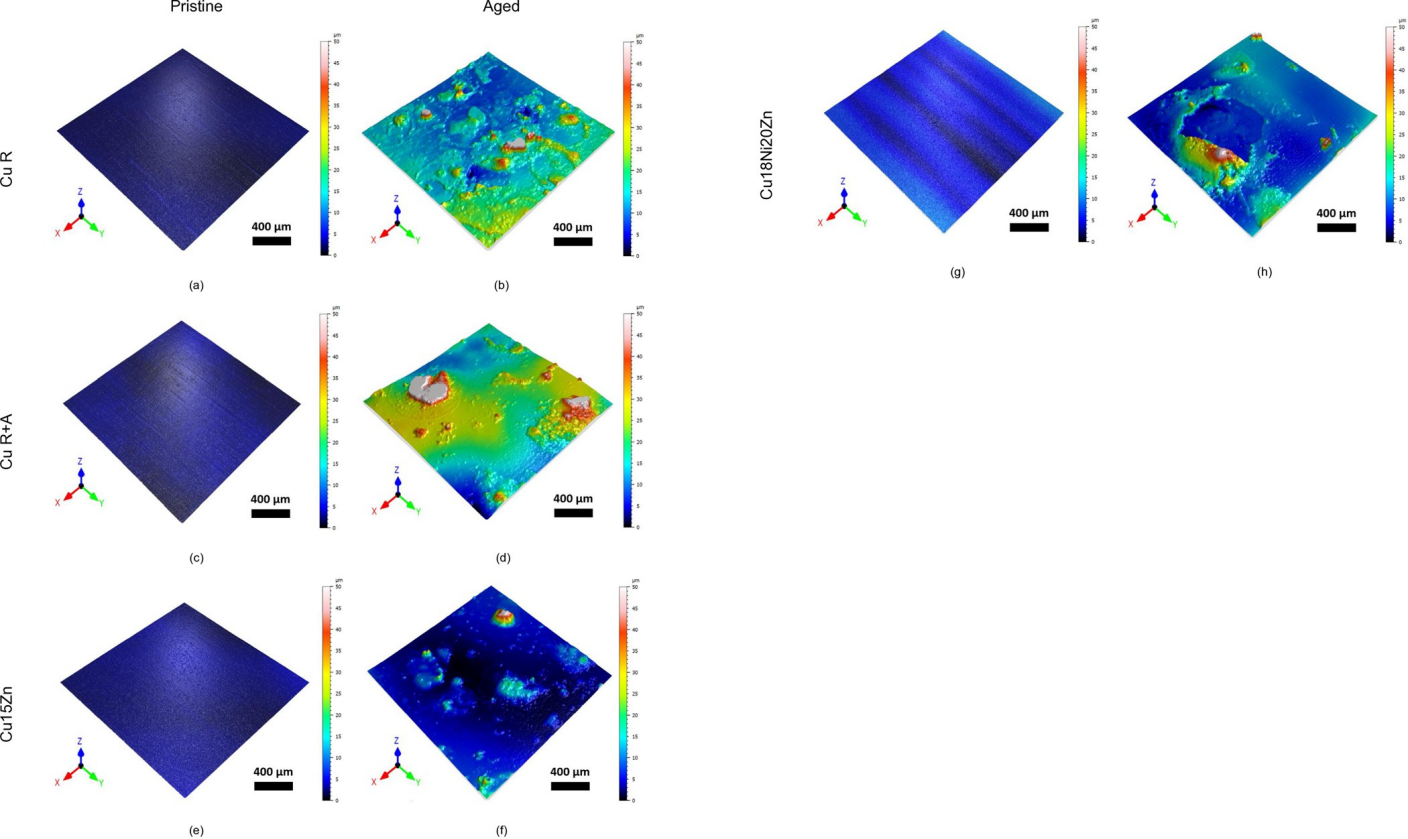
Surface topography of the Cu-based foils before and after accelerated ageing: (a) Cu R, (b) aged Cu R, (c) Cu R+A, (d) aged Cu R+A, (e) Cu15Zn, (f) aged Cu15Zn, (g) Cu18Ni20Zn, (h) aged Cu18Ni20Zn.

The development of corrosion products was confirmed by noticeable topographical changes (Fig 6), characterized by a more or less homogeneous distribution of circular aggregates. This may be due to the tendency of the ASW liquid droplets to coalesce after spraying, leading to radially generated corrosion products, as also observed by [19]. These localized corrosion products tend to form cracks, detectable in all the high magnification SEM images of Fig 5C, 5F, 5I and 5L as well as in the topography of Fig 6H.

The highest Δm % value was measured on Cu18Ni20Zn, followed by Cu R, Cu R+A, and Cu15Zn. However, the brittleness of corrosion products makes correlations between alloy type and Δm% values less significantly relevant. More valuable information comes from optical images in Fig 5 and EDS data in Table 3, showing that, after ageing, Cu R is covered by the most continuous layer of corrosion products and shows the highest O element wt.% increase, whilst Cu18Ni20Zn is covered by the smallest corrosion spots and undergoes the lowest O increase. In the former case, the low corrosion resistance of Cu R is due to its strain-hardened microstructure (not compensated by final annealing), as indicated by microstructural data derived in our previous work [31] as well as by the absence of alloying elements which may favour the formation of passive layers, whilst in the latter case the recrystallized microstructure of Cu18Ni20Zn (induced by annealing) combined with significant alloy additions (particularly Ni [44]) contribute to improve corrosion behaviour.

**Fig 7:**
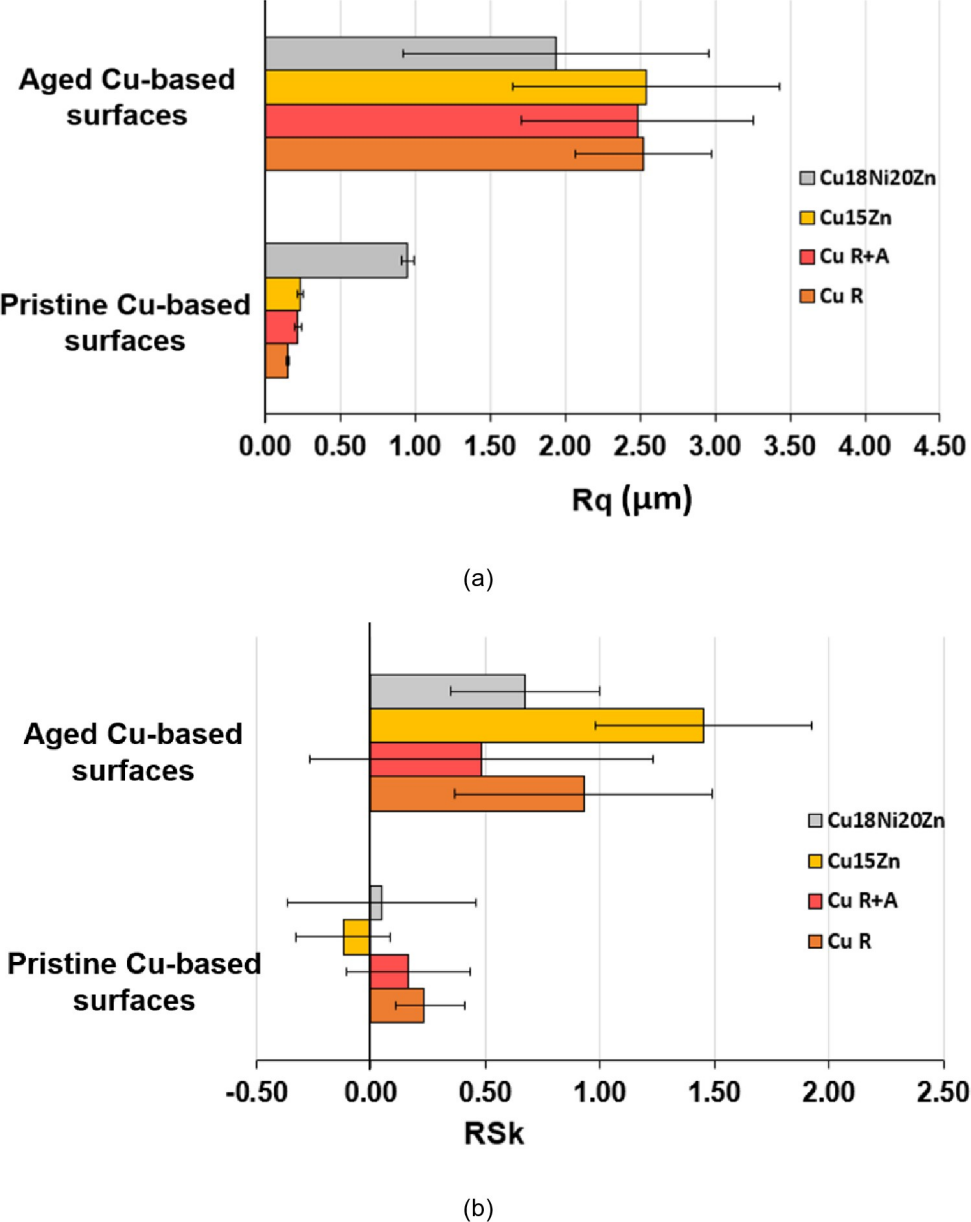
Surface roughness parameters of the Cu-based thin foils before and after accelerated ageing: (a) root mean square surface roughness (Rq) and (b) skewness (Rsk).

Fig 7 shows surface topography parameters (root mean square roughness (Rq) in Fig 7A and skewness (Rsk) in Fig 7B) measured before and after ageing. These parameters are known to influence surface wettability, which plays a crucial role in determining bacterial adhesion and, hence, the antimicrobial capacity of the surface [45]. In this work, a visible dilation of the drop was observed once it touched the Cu-based surface. This was particularly evident on aged surfaces such as Cu18Ni20Zn. Indeed, a significant increase in Rq after ageing was observed due to the formation of the previously described corrosion products. The % variation of Rq due to ageing (ΔRq%) follows the same trend already discussed on the basis of morphological and elemental composition data (Fig 5 and Table 3): Cu18Ni20Zn (104%) < Cu15Zn (967%) < Cu R+A (1037%) < Cu R (1577%), showing that Cu18Ni20Zn underwent the lowest surface modification due to corrosion and Cu R the highest. It is also worth noting that Cu18Ni20Zn showed the highest Rq value before ageing due to the lowest thickness of this foil, which consequently underwent a significant extent of wrinkling.

Similarly to Rq, also Rsk increased after ageing (Fig 7B), indicating an alteration of the peak-to-valley distribution. Namely, Rsk values were small and near zero before ageing, indicating a random distribution of peaks and valleys in pristine foils. After ageing, Rsk increased and achieved positive values for all samples. Rsk > 0 is associated with a predominance of peaks over valleys [46] due to the formation of the localized corrosion products shown in Figs 5 and 6. Cu15Zn exhibited the highest increase in Rsk, indicating that localized corrosion products were shaped as relatively high “bubbles” (Fig 5I).

Colour variation is reported in Table 4. The ageing process led to a significant decrease both in the L* parameter (darkening) and in a* and b* coordinates (tendency to green-blue, typical colour of Cu-based corrosion products, as also shown by optical images in Fig 5), for all surfaces. Cu R displayed the highest darkening and colour variation after ageing, followed by Cu15Zn, Cu R+A and Cu18Ni20Zn, which showed the most negligible darkening and colour change. The same trend was observed for ΔE values, which represent the total colour variation. The low tendency of Cu18Ni20Zn to surface alteration, already highlighted by previous results, is likely due to the improvement of corrosion resistance due to Ni, as reported by Fredj et al. [47].

**Table 4:** Variation of a*, b* and L* between aged and pristine Cu-based surfaces (CIELab color space).

	ΔL*	Δa*	Δb*	ΔE
Cu R	-36.10	-5.15	13.28	38.81
Cu R+A	-26.25	-1.33	-0.33	26.29
Cu15Zn	-32.47	0.47	-9.22	33.76
Cu18Ni20Zn	-1.57	-1.15	-1.24	2.31

The darkening of most surfaces (except the less corroded surface, i.e. Cu18Ni20Zn) is related to the formation of corrosion products, as shown by optical images in Fig 5B, 5E, 5H and 5K. Even though the surface of aged foils was analysed by micro-Raman spectroscopy, it was not possible to identify the corrosion products due to their low thickness as well as their low degree of crystallinity. Corrosion products detected on aged Cu-based surfaces typically consist of reddish/dark-brown cuprous oxide (Cu_2_O, Cu (I)). At the same time, green-blue colour changes can derive from the development of Cu (II) products such as Cu chlorides (i.e. CuCl) and oxychlorides (i.e., Cu_2_Cl(OH)_3_), as well as carbonates (i.e. CuCO_3_), which are consistent with modifications of elemental composition reported in Table 3, and were detected in similar conditions after more prolonged ageing by [19, 48]. In fact, Cu_2_O is the first product to form on a dry Cu-based surface in ambient air and humidity conditions [49]. In the presence of Cl^-^ ions, such as during ASW cyclic exposure, the formation of Cu_2_O is further favoured, as also observed in the literature on artificial sweat mediate-corrosion of Cu and Cu alloys [47, 50]. In the case of this work, the higher increase in O, C, Cl % wt.% (Table 3) in Cu R, Cu R+A and Cu15Zn, combined with more evident darkening (Table 4) of these surfaces compared to Cu18Ni20Zn indicates a more advanced degree of corrosion, likely involving the development of Cu_2_O or soluble Cu^+^ salts (alongside more complex corrosion compounds), which generally play a key role in antimicrobial activity (Cu^+^ dissolved ions are generally considered more toxic than Cu^2+^ [51]).

### 3.2 Biofilm inhibition assessment against *P*. *aeruginosa*

**Fig 8:**
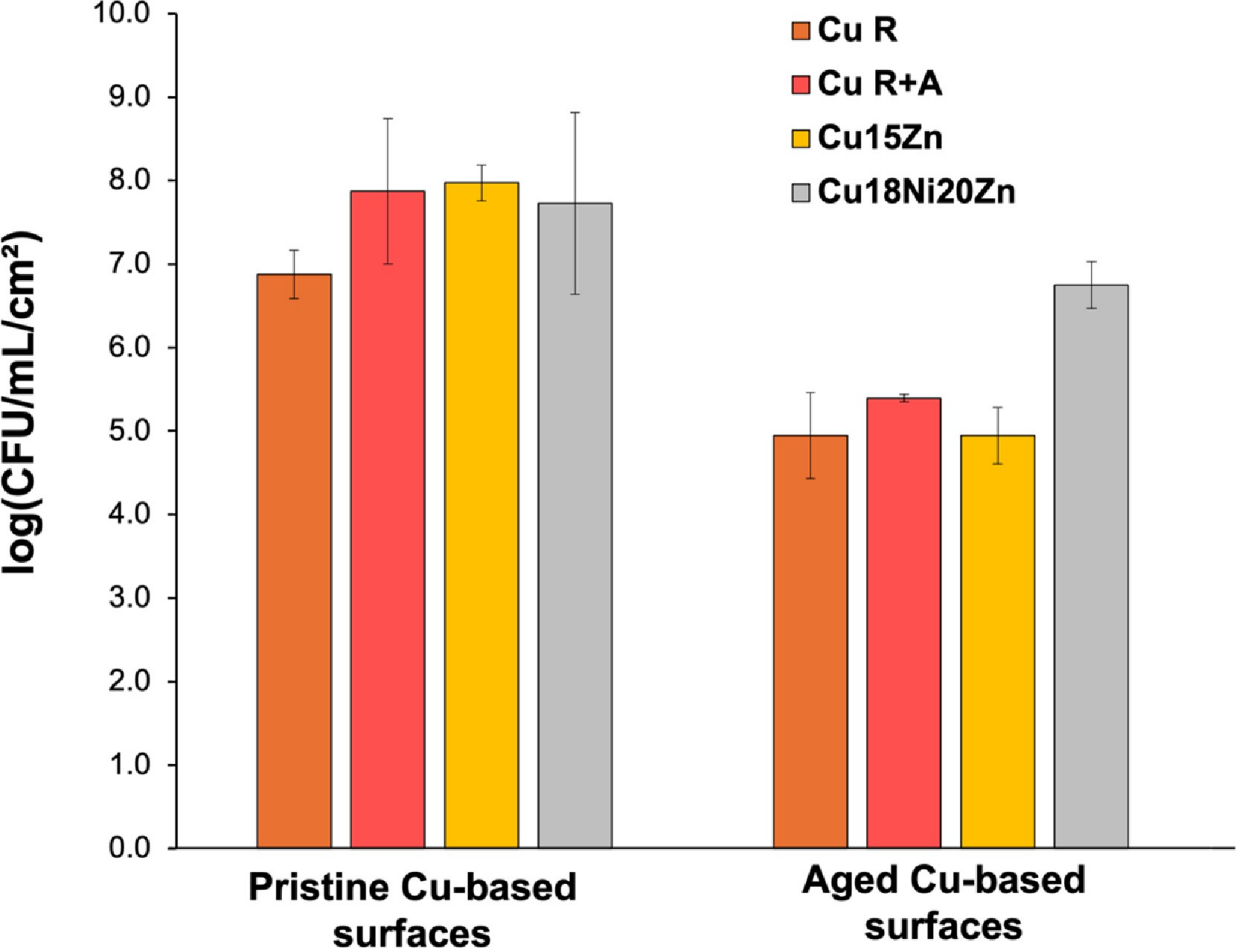
Biofilm formation (CFU/mL per unit surface area (cm^2^)) of *P*. *aeruginosa* on Cu-based surfaces (pristine vs. aged).

Fig 8 shows biofilm formation data for both pristine and aged samples. The nature of our experiment does not distinguish between the ability to attach and the killing of attached cells. The microbiology experiments reveal a strong decrease in biofilm formation capacity on aged Cu alloy surfaces compared to each pristine counterpart. The only exception was Cu18Ni20Zn, which displayed comparable values between pristine and aged conditions. The biofilm formation capacity decreased due to ageing as follows Log_10_ (CFU/mL/cm^2^ for aged surfaces)—Log_10_ (CFU/mL/cm^2^ for pristine ones): Cu18Ni20Zn (-0.98) < Cu R (-1.93) < Cu R+A (-2.48) < Cu15Zn (-3.03). It is important to mention that a 3 Log_10_ reduction in CFU is typical of a strong antimicrobial effect as reflects a 99.9% efficacy equivalent to many antiseptics on the market.

**Fig 9:**
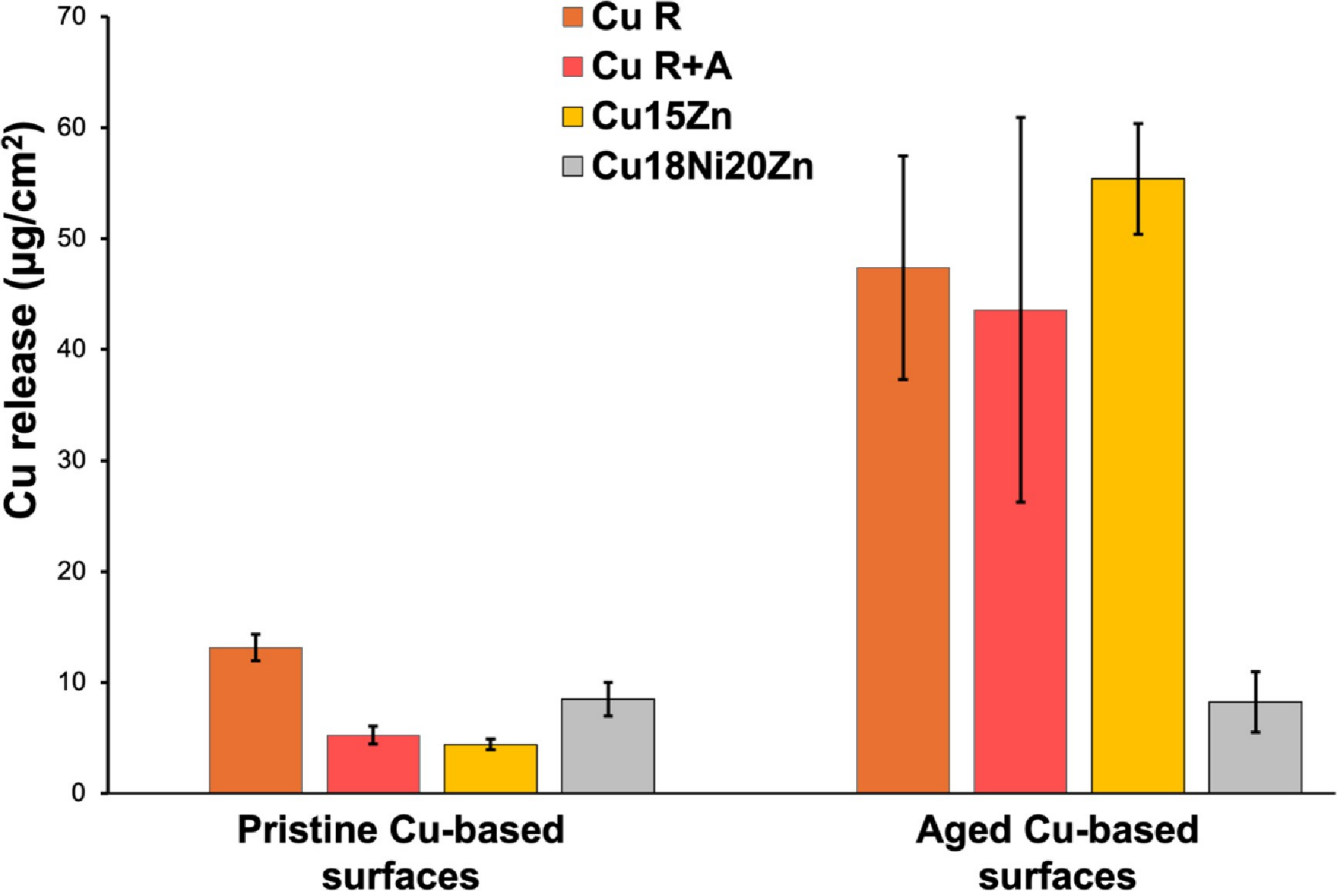
Cu ion release in LB droplets after 30 min of exposure in semi-dry conditions.

The decrease of *P*. *aeruginosa* biofilm formation/stability is correlated to ageing and the trend of increase of Cu ion release (Fig 9).

Specifically, Cu release (expressed as the aged/pristine ratio) increased as follows: Cu18Ni20Zn (1.0x) < Cu R (3.6x) < Cu R+A (8.3x) < Cu15Zn (12.6x). Also for Cu release, a similar trend as for biofilm formation was observed; the exception was Cu18Ni20Zn, which maintained comparable Cu release values regardless of the surface condition (i.e., pristine or aged). In fact, the t-test analysis highlighted that only the Cu18Ni20Zn pristine-aged couple did not provide statistical differences in Cu release values.

In Table 5, the main outcomes are summarized, in accordance with the flowchart (Fig 1).

**Table 5:** Summary of the main outcomes of this study.

Investigation type	Main Outcomes
**Surface characterization**	Localized corrosion on all the surfaces after the ageing sequence.
	Cu R covered by the most continuous layer of corrosion products and shows the highest O element wt.% increase, due to its strain-hardened microstructure.
	Cu18Ni20Zn is covered by the smallest corrosion spots and undergoes the lowest O increase thanks to its recrystallized microstructure and the presence of alloying elements (i.e., Ni) that enhance its corrosion resistance.
	% variation of Rq: Cu18Ni20Zn (104%) < Cu15Zn (967%) < Cu R+A (1037%) < Cu R (1577%).
	Cu15Zn exhibited the highest increase in Rsk, indicating that localized corrosion products were shaped as relatively high “bubbles”.
	Color variation (ΔE) after ageing: Cu R > Cu15Zn> Cu R+A > Cu18Ni20Zn.
**Biofilm inhibition assessment against P. aeruginosa**	Strong decrease in biofilm formation capacity on aged Cu alloy surfaces compared to each pristine counterpart.
	Biofilm formation capacity Log_10_ (CFU/mL/cm^2^ for aged surfaces)—Log_10_ (CFU/mL/cm^2^ for pristine surfaces):
	Cu18Ni20Zn (-0.98) < Cu R (-1.93) < Cu R+A (-2.48) < Cu15Zn (-3.03)
**Cu release**	Cu release variation due to ageing: Cu18Ni20Zn (1.0x) < Cu R (3.6x) < Cu R+A (8.3x) < Cu15Zn (12.6x).
	Among pristine surfaces: Cu R > Cu18Ni20Zn > Cu R+A ≃ Cu15Zn
	Among aged surfaces: Cu15Zn ≃ Cu R ≃ Cu R+A > Cu18Ni20Zn

The direct correlation between the decrease of biofilm formation and the increase of Cu release after ageing highlights the key role of Cu ions in *P*. *aeruginosa* biofilm inhibition.

Among aged surfaces, statistical differences were found for Cu release between Cu18Ni20Zn and the other Cu-based surfaces (Cu R, Cu R+A and Cu15Zn). For aged surfaces, the differences in Cu release and hence the different ability to inhibit biofilm formation can be explained based on the previously discussed corrosion-related surface features (Figs 5 and 6 and Tables 3 and 4), considering that Cu ion may dissolve from corrosion products [48].

Cu18Ni20Zn showed the lowest surface modification due to corrosion, hence, Cu release here may not be activated by corrosion, thus leading to the lowest decrease of biofilm formation after ageing. Zn and Ni were actually reported to reduce the amount of Cu ion released in the same artificial perspiration solution used here, due to lower corrosion rates coupled with preferential Ni and Zn oxidation [48]. An alternative at this time that cannot be ruled out is that cells could not adhere to the less corroded and less roughed surfaces as effectively [52]. Cu R displayed a slightly higher decrease of biofilm formation after ageing than Cu18Ni20Zn, due to its lower corrosion resistance hence higher Cu release. Cu R+A was slightly more corrosion-resistant than Cu R (thanks to final annealing, which relieved strain hardening). Therefore, the more discontinuous corrosion layer in Cu R+A was less effective than in Cu R for limiting Cu release, hence leading to higher anti-biofilm efficiency.

Cu15Zn is more corrosion resistant than unalloyed Cu and showed the highest decrease in biofilm formation after ageing. This is probably due to the formation of even more discontinuous corrosion products, unable to limit Cu ion release after ageing, maximising the detrimental effects on biofilm.

On aged surfaces, despite an increase in drop area allowing an increment in cell attachment, a decrease in biofilm was quantified. This suggests that the higher Cu release was likely effective in reducing any additional cell attachment.

For pristine samples, the highest biofilm inhibition capacity was observed on Cu R, whilst the other surfaces displayed statistically comparable values. This result is again associated with the highest Cu release, which in Cu R was induced by the higher reactivity of the strain-hardened microstructure in ASW, further highlighting the direct correlation between biofilm inhibition and Cu release.

Among pristine, statistical differences for Cu release were found between each surface type, except for the Cu R+A and Cu15Zn couple. For pristine surfaces, Cu release may also be related to surface roughness. It has been demonstrated that rough surfaces exhibit a higher Cu ion release rate over time than smooth ones [51], thus showing greater antibacterial activity in a medium composed of 0.9% NaCl [53]. However, our study did not show a significant impact of surface topography on the antibiofilm activity of pristine samples. Cu18Ni20Zn, which showed the highest roughness before ageing (Fig 7A), did not display appreciable differences in antibiofilm capacity (Fig 8) compared to the other pristine surfaces, apart from Cu R (where microstructural factors play a dominant role, as previously commented). The low antibiofilm capacity of pristine Cu18Ni20Zn compared to other pristine Cu-based foils may be more likely attributed to other factors: (i) lowest Cu content and (ii) lowest coverage of the surface by corrosion products from which Cu ions can dissolve [48]. Therefore, the low concentration of Cu ions on the surface can lead to the stratification and stabilisation of biofilm within the drop, generating less detrimental conditions for biofilm initiation.

Indeed, it has been recognized by Werner *et al*., [54], that *P*. *aeruginosa* can develop stratified patterns of protein synthesis and growth in biofilms with also the possibility of finding them in inactive and non-dividing or metabolizing forms. Specifically, in the case of *P*. *aeruginosa* PA01 biofilms exposition to subinhibitory levels of Cu, cell mortality predominantly occurred at the outer surface and periphery of the biofilm, regions more susceptible to Cu exposure [17, 55]. Thus, a gradient in the spatial distribution of viable cells has been noted within *P*. *aeruginosa* PA01 biofilms when exposed to subinhibitory concentrations of Cu [55]. It can especially occur on pristine substrates, beyond aged Cu18Ni20Zn. In a situation where biofilm is exposed to toxic compounds, and generally, in a mature biofilm, steep gradients of any kind of parameters such as antimicrobial metals (i.e., Cu) occur. Within the biofilm structure, Cu ions experience diffusion-reaction inhibition within the biofilm matrix as it can be complexed by polysaccharides, [56], and proteins such as siderophores, impeding its diffusion into the biofilm matrix [57]. Specifically, *P*. *aeruginosa* possesses the ability to chelate Cu^2+^ ions within the biofilms extracellular matrix (ECM) [58] via ionic interactions with negatively charged carboxylate or phosphodiester groups. Moreover, metal cations in biofilm ECM may also undergo covalent reactions with sulfates, thiolates, and phosphates [58]. These functional groups are widely present, indeed, carboxyl groups belong to acid polysaccharides, phosphoric acid groups to phospholipids and sulfhydryl to metal-binding proteins like metallothionein [59]. Lastly, given that proteins constitute a substantial portion of the ECM, it is widely acknowledged that biofilms react to metal toxicity by inducing metabolite production to facilitate protein synthesis. This alteration in the protein composition and amount within the biofilm enhances the biofilm’s defensive capabilities [60].

As mentioned above, the Cu’s antifouling properties observed in this work appear to be primarily attributed to the release of Cu ions. This can cause cell damage by altering protein and enzyme structure and activity, and biofilm breaking down by the attack of Cu. Specifically, Cu breaks biofilm by attacking ECM, inducing ROS production and binding QS-signalling molecules, ultimately determining the disruption of QS and biofilm inhibition [13, 61]. Indeed, bacterial cells and biofilm are connected via QS since biofilm structure is regulated by QS, based on the production of autoinducer molecules, which depends on cell density [62]. In *P*. *aeruginosa*, the molecular mechanism of QS involves three primary pathways: las, rhl, and pqs [63]. The QS related to biofilm structure comprises the LasI and LasR proteins that regulate biofilm polysaccharide production, which defines biofilm structure formation [64]. QS can be suppressed by Cu ions, resulting in biofilm formation inhibition. Indeed, Mishra *et al*., [65] demonstrated that Cu ions via Cu nanoparticles may cease QS signalling by binding to alkyl-homoserine lactone (AHL) synthases (LasI/RhlI), thereby interfering with AHL production. Additionally, they can bind to receptor proteins (LasR/RhlR), potentially preventing the activation of transcriptional activators.

The main limits and strengths of this study can be summarized as follows:

-Limits: (i) the choice of culture media such as LB, which is required for sustaining the growth of *P*. *aeruginosa*, may not fully represent a high-touch situation, where the medium mostly consists of human sweat; and (ii) not using more severe ageing conditions or longer-term exposure. However, these ageing conditions represent the mild deterioration which may occur between cleaning cycles of Cu-based surfaces in a healthcare setting.
-Strengths: (i) use of a widely applied medium (LB), used in several works to investigate antimicrobial and antibiofilm properties [66–68], which maximises the bacterial adhesion on surfaces as worst case scenario since Macomber *et al*., [69] found that nutrient-rich media such as LB increased the bacterial tolerance to high Cu levels. Moreover, LB, due to its high aminoacid concentration, promotes flat and uniform biofilm formation [70]; (ii) use of an exposure time (30 min) which has been defined as satisfactory to allow bacterial attachment [71], (iii) use of accelerated ageing conditions representative of healthcare facilities, (iv) identification of microstructure- biofilm inhibition relationship for Cu-based foils with different alloy compositions in pristine and aged forms, derived within the same protocols.

## 4 Conclusions

This study examines the impact of simulated ageing on the *Pseudomonas aeruginosa* antibiofilm capacity of industrially manufactured Cu-based thin foils, intended for use in healthcare facilities by cladding onto high-touch surfaces. Accelerated ageing of the Cu-based surfaces was carried out in conditions mimicking repeated human touch as well as humidity and temperature conditions of hospital environments. Antibiofilm capacity was assessed before and after ageing by exposing Cu-based surfaces with drops of LB with *P*. *aeruginosa* and correlated with alloy composition, microstructure, surface features and Cu release in LB medium solution, measured by semi-dry conditions. The following conclusions can be drawn from this work:

- Biofilm inhibition capacity is correlated with Cu release: ageing tends to trigger Cu release, hence decreasing biofilm stability. The extent of Cu release augmentation due to ageing depends on alloy composition and microstructure.
- The Cu release is affected by alloy microstructure: namely, strain-hardening increases Cu release and, hence, biofilm inhibition. Conversely, alloy additions may make biofilm inhibition capacity independent from ageing if the alloy is corrosion-resistant enough to be only slightly modified by ageing (as for Cu18Ni20Zn). Alternatively, alloy additions may increase antibiofilm capacity (as for Cu15Zn) if the morphology and distribution of corrosion products do not hinder Cu release.
- The increment in drop area after ageing does not lead to biofilm increase. From pristine to aged surfaces (except for Cu18Ni20Zn), where the increase in drop area occurred, potentially bringing to a more colonisable surface by bacteria attachment, a biofilm formation decrease was detected. Therefore, the higher Cu release was likely responsible for reducing any additional cell attachment.
- In the case of pristine alloys, as for both forms of Cu18Ni20Zn (pristine and aged), the lower tendency to anti-biofilm formation can be due to lower coverage of the surface by corrosion products from which Cu ions can dissolve and partly as a consequence of its lower Cu content. This leads to low Cu ion concentration within the drop which can lead to the stratification and stabilisation of biofilm.
- Cu15Zn could be the most effective surface in preventing *P*. *aeruginosa* biofilm formation in healthcare settings and crowded indoor environments. It follows PHC Cu, while Cu18Ni20Zn could be excluded.

## Supporting information

S1 File (XLSX)
